# Digging the metabolic roots of NASH up

**DOI:** 10.1093/lifemeta/loac036

**Published:** 2022-12-06

**Authors:** Enrica Baldelli, Amedeo Lonardo

**Affiliations:** Department of Biomedical, Metabolic and Neural Sciences, University of Modena and Reggio Emilia, Modena, Italy; Azienda Ospedaliero-Universitaria di Modena—Department of Internal Medicine—Ospedale Civile di Baggiovara, Modena, Italy

**“Maybe you are searching among the branches for what only appears in the roots”—Rumi.**

Substantiating the paradigm of disease heterogeneity, nonalcoholic fatty liver disease (NAFLD) has a remarkably variable natural history in the individual patient which legitimates personalized medicine approaches. Why some individuals follow a benign indolent course while others progress from simple steatosis to ongoing liver fibrosis, cirrhosis, and hepatocellular carcinoma (HCC) remains a fundamental (though unanswered) clinical and research question. In this context, although it is hepatic fibrosis, not nonalcoholic steatohepatitis (NASH), that dictates the prognosis and the clinical course of NAFLD, NASH is recognized as the critical link triggering the deterioration toward the most severe hepato-histological pictures in a proportion of NAFLD patients.

NASH defines typical histological connotations (steatosis + ballooning + mild inflammation) observed in individuals without any competing causes of chronic liver disease. Therefore, NASH diagnosis requires a clinico-laboratory work-up and liver biopsy, an invasive procedure potentially associated with untoward effects. These undesired effects, together with procedural costs, inter-observer variability of histological lesions, and patchy distribution of liver histology changes, collectively explain why researchers actively investigate noninvasive biomarkers of NASH [1]. Identifying and validating such biomarkers is important given that, in many areas of the world, NASH is a major driver of NAFLD progression to decompensated cirrhosis, HCC, liver transplant, as well as major extrahepatic complications.

**Figure 1:**
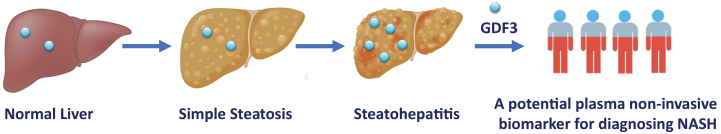
GDF3 is a potential noninvasive biomarker of NASH.

With this clinico-epidemiological backset, Xiang *et al.* [2] investigated genes, noncoding RNAs, proteins, and plasma metabolites, a strategy collectively named “comprehensive multi-omic profiling”, specifically addressing the fundamental molecular mechanisms governing the progression from steatosis to NASH (Fig. 1). To this end, the study included both an experimental section on mice and a clinical arm. Overall, this paper has three major findings of potentially diagnostic, pathogenic, and therapeutic significance.

First, transcriptomic analysis in mice identified growth differentiation factor 3 (GDF3) as a candidate noninvasive biomarker in NASH. Confirming this notion, in NASH patients, plasma GDF3 concentrations provided a fairly accurate diagnosis of NASH (AUROC = 0.90; 95% confidence interval: 0.85−0.95) with good values of sensitivity and specificity (90.7% and 86.4%, respectively). The major sources for GDF3 expression in the liver are macrophages and Kupffer cells [2]. Interestingly, GDF3 was also shown to mediate the critical crosstalk between macrophages and adipocytes in adipose tissue, eventually triggering adipogenesis and local and systemic inflammation in the setting of lipid overload-associated hyperinsulinemia owing to insulin resistance [3]. Thus, GDF3 may play a diverse role in steatosis-to-NASH progression. Besides, further studies are needed to confirm the diagnostic value of GDF3 across various patient ethnicities. With this limitation in mind, GDF3 is a fully plausible candidate biomarker of NASH, given that GDF3 is centrally positioned at the crossroad of obesity, insulin resistance, and inflammation, and is a major regulator of energy balance acting as an adipogenic factor in conditions of lipidic overload.

Second, Xiang *et al*. [2] have implicated ferroptosis as a potential pharmacological target in NASH progression based on integrated proteomic-metabolomic analysis and confirmed with a subsequent proof-of-concept drug intervention.

Ferroptosis is a non-apoptotic iron-dependent variety of cell death that typically includes free iron accumulation, oxidative stress, and degeneration of cell membranes induced by lipid oxidative damage [4]. All these three pathomechanisms reportedly occur in NASH. To illustrate the role of iron, a study conducted in 849 participants in the NASH Clinical Research Network [5] found that three histological subtypes of stainable hepatic iron could be observed based on the pattern of iron distribution: a hepatocellular (HC) variant (7.4% of cases), a reticuloendothelial system (RES) cell distribution (10.7% of cases), and a mixed RES/HC pattern (totaling 16.4% of cases). Alarmingly, the RES iron-staining pattern was independently associated with advanced hepatic fibrosis [Odds Ratio (OR) = 1.60, 95% confidence interval = 1.10−2.33, *P* = 0.015]. These findings support the notion that iron overload in the RES may be a cofactor promoting the progression of NASH via the Fenton reaction, lipid peroxidation, severe cell and tissue damage owing to unbuffered free radicals, inflammatory response, and progressive hepatic fibrosis [4, 5].

Metabolic disease may be conceptualized as occurring owing to disordered cellular iron distribution. Supporting this notion, NAFLD and NASH exhibit a high secretion from hepatocytes of iron-repleted extracellular vesicles which are cleared by Kupffer cells, explaining the apparent paradox of iron-deficient hepatocytes (which potentiates hepatocyte lipogenesis and insulin resistance through HIF2α−ATF4 signaling) living together with iron-overloaded HSCs (with overproduction of profibrogenic ROS). Consistently, drug interventions either blocking the secretion of extracellular vesicles from hepatocytes or depleting extracellular vesicles iron cargo will restore hepatic iron homeostasis and improve NAFLD/NASH liver histology [6].

Seemingly in conflict with the above data, a meta-analytic review has demonstrated that, compared to lifestyle changes, iron depletion fails to significantly improve insulin resistance, liver enzymes, and liver histology among those with NAFLD [7]. However, Hepcidin, a master regulator of iron homeostasis that can remove liver iron stores, was indeed able to suppress steatohepatitis and ensuing liver fibrosis in a mouse model of diet-induced NASH [8]. Therefore, additional investigations are warranted on this hot topic.

Studies have shown that that the severity of liver steatosis is associated with eight ferroptosis-related genes, and ferroptosis is at the crossroad of iron and lipid metabolisms, as well as intracellular redox homeostasis, tilting the balance toward the pro-inflammatory state at the onset of NASH [4]. High-fat diet induced increases in the intrahepatic concentration of the pro-inflammatory precursor arachidonic acid go in parallel with development and severity of NAFLD inflammatory changes. Closing the pathogenic circuit, inflammation also perpetuates ferroptosis, and inflammation and ferroptosis mutually complete each other [4].

Three parallel anti-oxidant systems are involved in ferroptosis, all of which are fueled by NADPH: Cyst(e)ine–GSH–GPX4 axis, NAD(P)H–FSP1–CoQ10 axis, and GCH1–BH4–DHFR axis, and these three metabolic pathways effectively inhibit the peroxidation of phospholipids, explaining why raised gamma-glutamyl transpeptidase values identify the most severe NAFLD forms and accounting for the rationale use of antioxidants (such as silymarin, silybin, or silibinin, pentoxifylline, resveratrol, and vitamins A, C, and E) and other drugs targeting ferroptosis to combat NASH [4, 9]. Future studies should address the interaction of intestinal dysbiosis with ferroptosis as an innovative therapeutic strategy in NAFLD (4).

Thirdly, Xiang *et al*. [2] reported that several microRNAs, including miR-582-5p and miR-292a-3p, and long noncoding RNAs, such as XLOC-085738 and XLOC-041531, were strongly associated with the steatosis-to-NASH progression. This finding implies that, by modulating gluconeogenesis and steatogenesis, and regulating the pro-inflammatory/anti-inflammatory balance, cell proliferation, metabolism, apoptosis, fibrosis, and carcinogenesis, variations of epigenetic expression are major modifiers of NAFLD initiation and progression across the entire disease spectrum from the stage of simple steatosis through cirrhosis and HCC [10]. On these grounds, epigenetic mechanisms are potential biomarkers, important disease cofactors, and also targets of therapeutic interventions in NAFLD arena.

In conclusion, the study by Xiang *et al.* [2] is a strong contribution to digging the metabolic roots of NASH up. This study proceeds towards further dissecting the mechanistic origins of disease pathogenic heterogeneity, while laying the foundations of more personalized medicine approaches in NAFLD arena. Future investigations will have to apply the above metabolic approach to liver fibrosis.
